# Supplementary figures and images for: Nocturnal hypercapnia with daytime normocapnia in patients with advanced pulmonary arterial hypertension awaiting lung transplantation
Source: PLoS One. 2020 Apr 15;15(4):e0227775. doi: 10.1371/journal.pone.0227775 (PMC7159234; doi:10.1371/journal.pone.0227775)

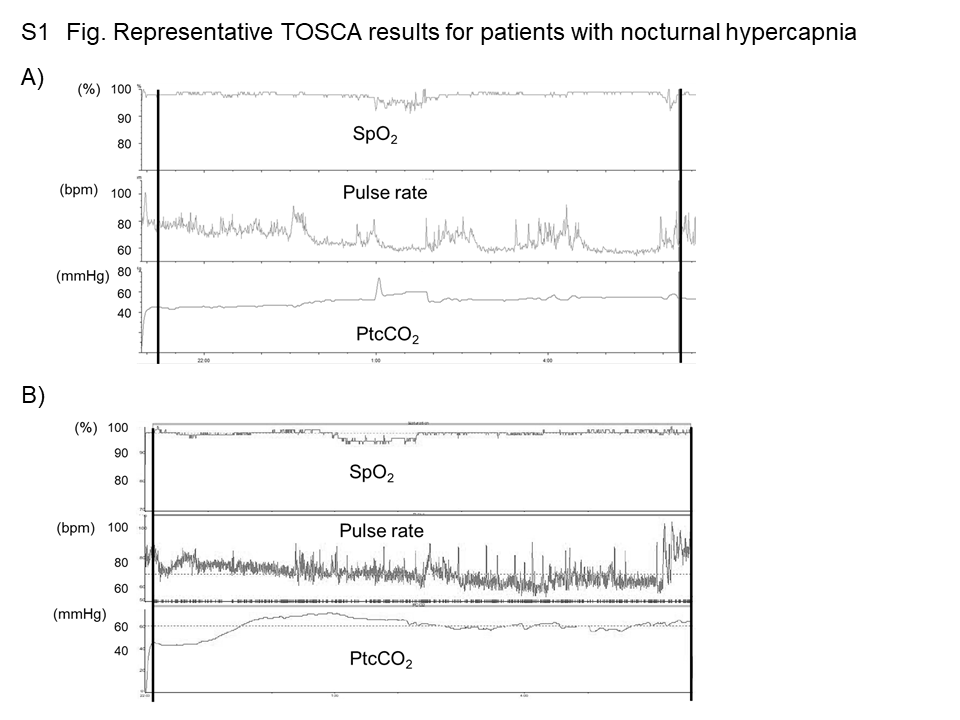

Supplement: S1 Fig — (A) TOSCA summary sheet for a 14-year-old female patient with IPAH and (B) 35-year-old female patient with IPAH. The intervals between the bold lines were analyzed. IPAH: idiopathic pulmonary arterial hypertension. (TIF) [file pone.0227775.s001.TIF]
